# The Role of Morphological Information in Processing Pseudo-words in Italian L2 Learners: It’s a Matter of Experience

**DOI:** 10.5334/joc.420

**Published:** 2025-01-07

**Authors:** Simona Amenta, Francesca Foppolo, Linda Badan

**Affiliations:** 1 Department of Psychology, University of Milano-Bicocca, p.zza dell’Ateneo Nuovo 1, 20126 Milan, Italy; 2 During the conceptualization of this work and data collection, Simona Amenta was affiliated with the Department of Experimental Psychology of Gent University, Gent, Belgium; 3 Department of Humanities, University of Trento, via Tommaso Gar 14, 38122, Trento, Italy; 4 During the conceptualization of this work and data collection, Linda Badan was affiliated with the Department of Translation, Interpreting and Communication, Gent University, Gent, Belgium

**Keywords:** novel derivations, pseudo-words, visual word recognition, L2 processing, morphology

## Abstract

The productive use of morphological information is considered one of the possible ways in which speakers of a language understand and learn unknown words. In the present study we investigate if, and how, also adult L2 learners exploit morphological information to process unknown words by analyzing the impact of language proficiency in the processing of novel derivations. Italian L2 learners, divided into three proficiency groups, participated in a lexical decision where pseudo-words could embed existing stems (e.g., *sock*le), suffixes (e.g., hett*able*), or both (novel derivations, e.g., *quickify*). Participants with low proficiency exhibited reduced accuracy and longer reaction times when presented with pseudo-words embedding a stem compared to those embedding a suffix. Conversely, participants with high proficiency demonstrated comparable accuracy in rejecting pseudo-words with real stems or real suffixes but required more time to reject pseudo-words embedding a suffix. In the case of novel derivations, accuracy (i.e., correct rejection) decreased and reaction time increased for all proficiency groups. Our results show that L2 learners exploit morphological information to process novel words. Most importantly, the ability to extract and exploit morphological information is linked to language proficiency.

## 1. Introduction

What is considered a word in a given language is a matter of agreement and use among speakers of that language. A few years ago, a story^1^ hit Italian media about an elementary school pupil who wrote the word “petaloso” (lit. many-petaled) on an essay. The teacher initially marked it as an error because the word is not included in the Italian lexicon. However, later, she wrote to the *Accademia della Crusca*, the Italian authority on the Italian language, asking for an opinion. A few days later the scholars of the *Accademia* wrote back saying that “petaloso” was indeed a well-formed word, it was understandable and interpretable and so it could be in principle be considered a word of Italian; however, it would be its use by Italian speakers to determine if the newly created word would enter the lexicon or not.^2^

What struck us about this story are some of the words used in the official response: the word *petaloso* could be a valid candidate to enter the lexicon because it was well formed in morphological terms (indeed the suffix –*oso* is legally attached to the stem *petalo*, a noun, to form a derived adjective), and because it could be understood by Italian speakers. This was a clear example of language productivity in action. Language productivity allows speakers to create new words and understand the meaning of words they have never heard or read before. At a sub-lexical level, morphology plays a fundamental role in the game of productivity, both in the process of novel word formation and novel word learning (e.g., Aronoff & Schvaneveldt, 1978; Frauenfelder & Schreuder, 1992; Günther & Marelli, 2020).

A construct that has gained growing attention among psycholinguists is indeed that of novel derived words (sometimes referred to as affixed pseudo-words, especially in the morphological processing literature) and their processing. A novel derived word is composed of an existing root and an existing suffix (e.g., *quickify* from *quick* + -*ify*), although the combination is not attested in the lexicon of the reference language (or in linguistic corpora of the same language). Morphological literature has first studied this type of pseudo-words in its very beginning, with the seminal work of Taft and Forster (1975). The authors were first interested in investigating whether morphologically complex words such as *learner* were decomposed in stem (*learn*) and affix (-*er*) during processing. Evidence from that first study showed that not only adult readers of English would decompose existing morphologically complex words in stem+ affix, but they would apply the same process also to affixed pseudo-words like “juvenate”, but not to pseudo-words with non-existing stems like “luvenate”.

Since then, several studies investigated the role of morphology in the processing of words and pseudo-words in a native language (L1) (see Amenta & Crepaldi, 2012 for a review). From the early stages of language acquisition, children are sensitive to word morphology and are able to use the morphemic constituents of words in comprehension and production tasks, particularly in languages with a rich inflectional system (e.g., Burani, Marcolini, & Stella, 2002; Duncan, Casalis, & Colè, 2009, among others). Previous research shows that children, from the early stages of reading exposure, learn to detect and exploit frequent and stable sequences of letters corresponding to morphemes, and use this ability to optimize fluency and accuracy in decoding unfamiliar words (Marelli, Traficante & Burani, 2020). For example, Burani et al. (2002) showed that morpho-lexical reading is available and efficient in young (and adult) readers of languages with a shallow orthography, like Italian (i.e., with regular grapheme-phoneme correspondence), and that the process extends to novel words presenting a morphological structure: both children and adults were faster in reading novel derivations, (e.g., donn*ista*), compared to non-morphological pseudo-words (e.g., denn*osto*).

A similar pattern has also been attested in a lexical decision task, suggesting an interference from the morphemic level in word lexical decision: Italian children were less accurate in rejecting novel derived words including an affix (e.g. *vetrezza*; Burani et al., 2002; Burani, Dovetto, Thornton & Laudanna, 1997). Similarly, English and French children were slower and less accurate in rejecting pseudo-words embedding a pseudo-stem and a suffix (e.g. *pondal*) in a lexical decision task, (Casalis, Quémart & Duncan, 2015). Similar results are reported by Dawson, Rastle and Ricketts (2018), where, again in a lexical decision task, children, young adolescents, older adolescents and adults were less accurate in rejecting pseudo-words embedding a pseudo-stem and an affix (e.g., *earist*) than pseudo-words embedding a pseudo-stem and a non-morphological ending (e.g., *earilt*). However, in this last study, only older adolescents and adults showed slower response latencies to morphological pseudo-words, suggesting a role of experience in the processing of these types of strings.

Converging evidence comes from morphological priming paradigms that used affixed pseudo-words as primes (e.g. *quickify*) for their embedded pseudo-stems (used as target; e.g., *quick*). In a masked priming lexical decision paradigm, Longtin and Meunier (2005) compared responses to prime-target pairs formed by either an interpretable affixed pseudo-word (*rapidifier*-RAPIDE) or an uninterpretable affixed pseudo-word (*sportation*-SPORT) or again a non-morphological pseudo-word (*rapiduit*-RAPIDE; where -*uit* is not a morpheme in French). The authors reported reliable priming effects for both affixed pseudo-words (e.g., *rapidifier* and *sportation*), but not for non-morphological pairs (e.g., *rapiduit*). Similar results were reported by Beyersmann, Casalis, Ziegler, and Grainger (2015) within the same paradigm and the same language (French); however, in this case, the authors controlled for the level of language proficiency of their L1 participants. As in the previous experiment, Beyersmann and colleagues compared lexical decision responses to prime-target pairs composed by suffixed pseudo-words (*tristerie*-TRISTE), non-suffixed pseudo-words (*tristald*-TRISTE) and suffixed words (*tristesse*-TRISTE). The authors reported equal priming effects for all three conditions for high proficiency participants; while participants in the low proficiency tier showed greater priming effects for suffixed conditions (independently of prime lexicality; e.g., *tristerie* and *tristesse*) compared to the non-suffixed condition (e.g., *tristald*). The authors conclude that while the results from the low proficiency group replicated the pattern found in Longtin and Meunier (2005), the results from the high proficiency group suggest that the activation of the embedded pseudo-stem is sufficient to boost the priming effect.

More recently, Hasenäcker, Beyersmann, and Schroeder (2016) compared lexical decision responses of children and adult speakers of German in a masked morphological priming task, where primes could be suffixed words (*kleidchen*-KLEID), suffixed pseudo-words (*kleidtum*-KLEID), non-suffixed pseudo-words (*kleidekt*-KLEID) or unrelated words (*traumerai*-KLEID). Results from the adult group showed reliable priming effect of the related conditions in comparison to the unrelated condition, and greater priming magnitude for the suffixed conditions in comparison to the non-suffixed condition. Children showed priming effect for the related conditions in comparison to the unrelated condition, but no difference between suffixed conditions and the non-suffixed condition. As we can assume that children have comparatively lower language proficiency than adults, the pattern reported by Hasenäcker et al. with German participants seem to go in the opposite direction of the results of Beyersmann et al. with French participants, where suffixed conditions generated greater priming effect in the low proficiency group in comparison to non-affixed pseudo-words.

Taken altogether, morphological priming literature on L1 speakers shows that young and adult speakers of a language use morphological information productively, as they are able to extract information from unfamiliar (novel) combinations of (pseudo-)stem and suffix: in all the studies reported above, participants experienced facilitation from primes which shared a pseudo-stem with the target and that also included an existing affix. Results about non-suffixed pseudo-words are less clear-cut as it seems that, in this case, language proficiency may exert an influence on how much the relative contribution of the embedded pseudo-stem over the affix weights in the processing of the pseudo-word itself. Therefore, some questions remain unanswered by previous literature on the processing of novel derivations, namely which is the relative contribution of stem and affixes, and which is the impact of language proficiency.

With respect to the studies on L1, the amount of research on the role of morphology in in a second language (L2) learning is still comparatively small. Generally, it has been demonstrated that L2 learners have difficulties in the application of morphological features (see for instance White, 2003). Behavioral research demonstrates that L2 learners acquire lexical items using decomposition and processing of inflections similarly to L1 speakers (Bosch & Clahsen, 2016; Portin, Lehtonen & Laine, 2007), even if with some differences (Farhy, Verissimo and Clahsen 2018; Kirkici & Clahsen 2013; Diependaele, Duñabeitia, Morris, & Keuleers, 2011, among others). It has been suggested that L2 learners may acquire sensitivity to the morphological structure with the increasing of language experience and proficiency (Diependaele et al., 2011). As for derived words processing, specifically for Italian L2, Dal Maso and Giraudo (2014) show that L2 learners increase their sensitivity to morphological information gradually with the increasing of the proficiency of their L2.

So far, research on L2 learners has mainly focused on existing morphology. However, this is not the condition in which L2 learners typically find themselves. On the contrary, on most occasions, L2 learners will confront words they have never read or heard before, and, most likely, these new words will be morphologically complex (e.g., Brysbaert, Stevenes, Mandera & Keuleers, 2016). For this reason, it is crucial to understand which cues are exploited by learners of a second language to recognize words and access their meaning.

Experimental paradigms using pseudo-words are extremely helpful in order to manipulate the degree of knowledge of lexical items (pseudo-words or novel words are *de facto* non-lexical items in a language) and the information fed to a reader. A few studies tackled this issue with respect to novel derived words (or affixed pseudo-words) reporting however contrasting results, especially when language proficiency is taken into account. In a simple lexical decision task, Casalis, Commissaire and Duncan (2015) compared responses of French (L1) – English (L2) speakers to affixed pseudo-words (*clockage*), pseudo-words formed by an existing stem and a non-morphological ending (*foodle*), pseudo-words formed by a non-existing stem and an existing suffix (*hettage*) and pseudo-words formed by non-existing stems and a non-existing suffix (*cottle*). The authors also considered the level of L2 proficiency dividing the participants in two groups. Results show that the presence of an existing stem (e.g., *foodle*) or an existing suffix (e.g., *hettage*) increased response latencies and decreased accuracy: in other words, it was more difficult for participants to reject as non-existing words, strings which included at least an existing stem or an existing suffix. The difficulty increased even more in the case of combination of a stem and an affix (e.g., *clockage*), that is, the case of novel derivations. No differences related to proficiency were reported in the L2 group, suggesting that L2 speakers were extracting and using morphological information to process the unfamiliar string independently of their proficiency.

Converging results are reported with the same paradigm by Li et al., (2017). In a simple lexical decision task, the authors compared responses of monolingual English L1 speakers and Chinese (L1)-English (L2 speakers) to affixed pseudo-words (*animalful*) and non-affixed pseudo-words (*animalfil*). Again, proficiency level of L2 speakers was controlled by dividing L2 participants in two groups based on their proficiency level. Results on the L2 speakers show that both sub-groups found it more difficult (both in terms of accuracy and response latencies) to reject affixed pseudo-words (e.g., *animalful*) in comparison to non-affixed pseudo-words (e.g., *animalfil*), indicating no role for language experience.

An effect of language proficiency was however reported by Kimppa, Shtyrov, Hut, Hedlund, Leminen & Leminen (2019) who ran a passive listening EEG experiment with L1 and L2 speakers of Finnish to compare the learning of derived words, inflected words, novel derivation and pseudo-suffixed words. They showed different ERP’s responses for novel derivation versus existing morphology only for advanced L2 and L1 speakers. No difference was found in L2 beginners, suggesting that the representations for complex form are not (yet) consolidated in early stages of learning.

In conclusion, if it is clear that morphemes play a key role in the processing, reading, learning and production of words and novel words, it is not yet clear how this role depends on language proficiency, as studies involving L2 failed in providing conclusive evidence over its impact. This is quite surprising since studies explicitly tackling morphological awareness development in L2 learners showed a progressive increase in morphological knowledge with language experience (e.g., Menut, Brysbaert & Casalis, 2022; Schmidtke, Rahmanian & Moro, 2022). This calls for further analyses as we would expect to see a clear effect of proficiency also in processing.

In this study we address this issue – focusing especially on language proficiency – by investigating the ability of native speakers of Dutch spoken in Flanders (Belgium), who study Italian as a L2, to use morphological information when reading novel derived words and pseudo-words embedding a morpheme (either a stem or a suffix). The general assumption is that relying on morphological cues might pose challenges in classifying as pseudo-words those items that contain known morphemes. With respect to previous research on this topic, we decided to treat proficiency in a more granular way considering three levels of proficiency instead of the two previously involved.

We address the following research questions: (i) Are L2 learners able to exploit morphological information during the processing of unknown words? (ii) Do L2 learners use morphological information *productively*?; (iii) Does the productive use of morphological information emerge with language proficiency? We hypothesize a positive answer to these questions, thus predicting: (i) that L2 learners will be able to identify and rely on morphological cues (i.e., suffixes) in the processing of known and unknown words and that (ii) they will experience increasing levels of difficulty in classifying novel derivations as pseudo-words in comparison to other types of pseudo-words (i.e., pseudo-words embedding *only* a stem or an affix); this suggests a productive use of morphological information. Furthermore, (iii) we expect the impact of morphological information to be modulated by language proficiency in the L2, with advanced learners being more influenced by the presence of morphemes.

## 2. Methods

### 2.1 Participants

We tested 95 native speakers of Dutch spoken in Flanders, who study Italian as L2, recruited at Ghent University and in two language schools for adult learning, Het Perspectif (Ghent) and SNT (Bruges), all located in Belgium. Participants were divided into 3 groups (beginner, intermediate, advanced, see Table 1 for further details)^3^ based on their level on the European Common Framework (CEFR).^4^ Fifty-one native speakers of Italian, all students at University of Milano-Bicocca (Milan, Italy), served as a control group.

**Table 1:** Characteristics of participants.


	CEFR LEVELS	NUMBER OF PARTICIPANTS	MEAN AGE (SD)^5^	FIRST LANGUAGE	MEAN YEARS OF EXPERIENCE WITH ITALIAN (SD)

Beginners	A1–A2	28	39.5(19.7)	Flemish	1 (0.17)

Intermediate	B1–B2	35	33.1(17.3)	Flemish	3.93 (2.2)

Advanced	C1–C2	32	35.5(21.8)	Flemish	7.94 (5.16)

L1	Native speakers	51	22.7(4.5)	Italian	


Of the L2 participants 73 identified as women and 22 as men. Of the L1 participants, 41 identified as women and 10 as men. None of the participants reported any cognitive or neurological problems and all of them had normal or correct-to-normal vision.

The experiment was carried out in compliance with the Helsinki Declaration and the ethical regulation of both Ghent University and the University of Milano-Bicocca. Before the beginning of the experimental session, participants were provided with informed written consent and the privacy management policy forms. All participants took part in the experiment on a voluntary basis, did not receive any compensation and gave written informed consent; L1 participants received course credits for their participation.

### 2.2 Materials and procedure

All four groups participated in a non-speeded lexical decision task where they had to decide if the string appearing on a computer screen was an Italian word or not.

The experiment material comprised 150 pseudo-word items divided in three categories (50 items per type). “Real Stem” strings were formed by an existing Italian stem^6^ plus a non-morphemic ending (e.g., *calzeccia*; *calz*- meaning sock); “Real Suffix” strings were formed by a non-existing “stem” plus an existing suffix (e.g., *tencapabile*; -*abile* meaning –able); finally “Novel Derivation” strings were formed by a novel legal combination of an existing stem plus an existing suffix (e.g., *stelloso*; interpretable as ‘with many stars’). All Novel Derivations were not attested in main Italian dictionaries (e.g., Treccani), nor in SUBTLEX-IT (Crepaldi, Amenta, Mandera, Keuleers & Brysbaert, 2015, a corpus of 128.339.065 million Italian words). We used 13 derivational suffixes (-*abile, -aggine, -eria; -zione, -ezza, -ismo, -iere, -ista, -itudine, -mento, -enza, -oso, -tore*) to create all complex pseudo-words; moreover, we selected 13 common non-morphemic word endings in Italian to create “Real Stem” pseudo-words. For the purpose of the paradigm, we also created 100 word items which served as fillers; 50 word items were morphologically complex and were created using the same suffixes used for pseudo-word items; 50 word items were instead morphologically simple and ended with the same non-morphemic endings as the Real Stem pseudo-words. The use of the same endings (morphemic or not) throughout the item set was aimed at limiting possible variability due to their eventual different relative frequencies. Item structure and their main psycholinguistic features are summarized in Table 2.

**Table 2:** Characteristics of the items included in the experiment. Each variable is accompanied by its average, standard deviation, and range (in brackets). In this table, we opted to use the term “Base” instead of “Stem” since the Italian words used as (pseudo)stems were not free-standing stems. Instead, we provide frequency and family size for the bound form that includes the inflectional affix (e.g., *calza*, Eng. *sock*, is formed by the root *calz-* and the inflectional affix *-a*, denoting singular feminine; this is considered the base form of the word). Item and base frequency values were obtained from Subtlex-IT (Crepaldi et al., 2015), and for both measures, we present Zipf transformed frequencies (Brysbaert et al., 2018). Suffix frequency, base family size, and suffix family size were calculated based on Subtlex-IT, considering all words with a frequency higher than 1. The complete itemset, including all measurements, as well as the script used for computation, can be found at: https://osf.io/mpfdy/?view_only=7bc2f6e47e4c4cd98e4065c475f901fd.


TYPE	EXAMPLE	STEM	SUFFIX	ITEM LENGTH	ITEM FREQUENCY	BASE FREQUENCY	SUFFIX FREQUENCY	BASE FAMILY SIZE	SUFFIX FAMILY SIZE

Real Suffix	Tencapabile	***	-abile(-able)	10.02 (1.41; 7–13)	NA	NA	5.48 (0.73; 3.83–6.41)	NA	529.88 (415.77; 30–1675)

Real Stem	Calzeccia	Calz-(Sock)	***	7.40 (1.14; 6–11)	NA	4.6 (0.72; 2.8–6.36)	NA	443.22 (613.26; 12–2573)	NA

Novel Derivation	Stelloso (Starful)	Stell-(Star)	-oso(-ous)	9.80 (1.56; 7–14)	NA	4.22 (0.73; 2.25–5.31)	5.46 (0.73; 3.83–6.41)	140.68 (233.14; 2–1344)	536.36 (412.47; 30–1675)

Complex Word	Amabile(Lovable)	Am-(Love)	-abile(-able)	9.98 (1.85; 6–14)	3.37 (1.08; 1–5.07)	4.245 (0.88; 2.19–6.36)	5.46 (0.71 3.83–6.41)	526.40 (2175.45; 3–15468)	514.88 (414; 30–1675)

Simple Word	Freccia(Arrow)	***	***	6.36 (1.52; 4–10)	3.6 (0.9; 1.2–6)	3.6 (0.9; 1.2–6)	NA	171.54 (462.4; 2–2740)	NA


All items were presented in randomized order using Psychopy2 Experiment Builder v1.85.2 (Peirce, 2007; Peirce et al., 2019).^7^ At the beginning of each trial, participants had to focus on a fixation point (a white *) appearing in the middle of the screen for 1s. The fixation mark was followed by a white, lower-case Courier New font 32 target, which stayed on screen until a response was produced. Participants were asked to respond as quickly and accurately as possible by pressing the J key if the item appearing on the screen was an existing word of Italian and the F key if it was not.

## 3. Data Analysis

Data were analyzed in the R environment (R Core Team, 2021) using R stats (version 4.0.0) and the packages lme4 (Bates, Maechler, Bolker, Walker, Christensen, Singmann, H., … & Grothendieck, 2011) and lmerTest (Kuznetsova, Brockhoff, & Christensen, 2017). The full dataset and analysis script is available at: https://osf.io/mpfdy/?view_only=7bc2f6e47e4c4cd98e4065c475f901fd.

### 3.1 Analyses of accuracy

**Figure 1:**
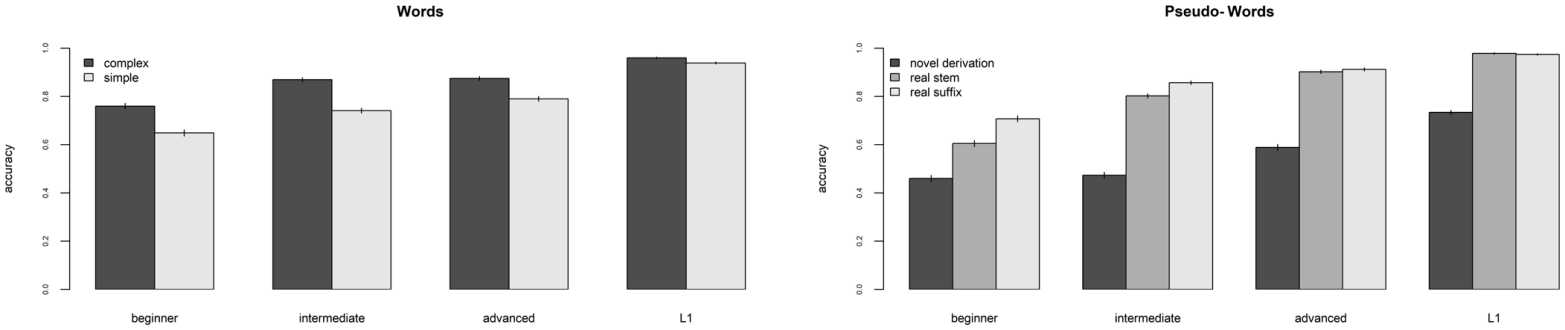
Mean accuracy rates for words (1A – left panel) and pseudo-words (1B – right panel) across word types and proficiency groups. Tables with detailed information is included in Appendix 1.1 and 2.1.

Figure 1 plots the accuracy rates for words and pseudo-words across types and groups.

First, we analyzed the accuracy performance of each group at the lexical decision task, when considering word targets, without distinguishing between simple and complex words. This served as a check of the correct execution of the task and implicit indication of the lexical proficiency of participants. Data were analyzed by fitting a logit mixed effects model (Jaeger, 2008), with *group* as predictor, log transformed word *frequency* and word *length* as covariates,^8^ and random intercepts for *items* and *participants* (to model variability related to items and subjects).^9^ We set a forward difference contrast coding scheme for the *group* variable (4 levels: Beginner, Intermediate, Advanced, L1; see Appendix 1.2 for the coding matrix).^10^

The full model of accuracy data for word targets is reported in Appendix 1.3. Frequency had a significant impact on accuracy (OR = 1.43, *z* = 8.51, *p* < .0001), while length did not (OR = 0.096, *z* = 1.64, *p* = .101). As expected, beginners’ performance was significantly lower than those of intermediate participants (OR = –0.82, *z* = –3.77, *p* < .001), while data showed no significant difference in the performance of intermediate and advanced participants (OR = –0.31, *z* = –1.45, *p* = .147). Finally, L1 participants showed significantly higher accuracy rates than advanced participants (OR = –1.997, *z* = –9.65, *p* < .001).

To account for the influence of morphological information, we tested the interaction between *group* and *type*. To do so, we first ran two logit mixed effect models: one with *group* and *type* as predictors, log transformed word *frequency* and *length* as covariates, and random intercepts for *items* and *participants* (our baseline model); and one with *group by type* interaction, log transformed word *frequency* and *length* as covariates, and random intercepts for *items* and *participants*.^11^ We compared the two models in terms of goodness of fit, that is we investigated if the model with the added interaction presented a better fit to the data than the baseline model. For each model we computed the Aikake Information Criterion (AIC; Akaike, 1973; Bozdogan, 1987) and the Bayesian Information Criterion (BIC; Schwarz, 1978). AIC and BIC are two commonly used metrics to compare statistical models and assess which one fits the data better: lower values of AIC and BIC indicate a better fit. Our comparison revealed that the interaction added significantly to the model fit (*χ^2^* = 11.003, *p* = .012; see Appendix 1.4 the full comparison and Appendix 1.5 for the estimates of the interaction model). We hence proceeded to explore the *group by type* interaction pattern within each group by means of planned comparisons. We hence ran four models – one for each proficiency group – with *type* as predictor, *frequency* and *length* as covariates, and random intercepts for *items* and *participants*. Results showed a significant effect of word frequency in all models (Beginner: OR = 0.83, *z* = 4.79, *p* < .001; Intermediate: OR = 1.92, *z* = 8.78, *p* < .001; Advanced: OR = 2.37, *z* = 8.30, *p* < .001; OR = 1.50, L1: *z* = 7.50, *p* < .001), while length was only a significant predictor for beginners (OR = –0.22, *z* = –2.53, *p* = .011) and advanced (OR = –0.28, *z* = –2.27, *p* = .023) participants. Results of word type indicated that morphologically complex words obtained significantly higher accuracy rates than morphologically simple words in all groups (Beginner: OR = –1.77, *z* = –4.11, *p* < .001; Intermediate: OR = –2.47, *z* = –4.85, *p* < .001; Advanced: OR = –2.48, *z* = –3.91, *p* < .001; L1:OR = –1.35, *z* = –2.87, *p* = .004), but the effect size was smaller for L1 participants, as it is also visible in Figure 1A (see Appendix 1.6 for the full model).

Next, we analyzed responses to pseudo-word items, which are the core of our study. We started with analyzing accuracy data, that is, the number of correct rejections, as plotted in Figure 1B. To compare accuracy scores for different types of pseudo-words we ran two mixed effects logit models and compared them using the same procedure detailed for word items. The first model included *group* and *type* as predictors, *length* as covariate and random intercepts for *items, participants*, and *string-endings*;^12^ the second model included the interaction *group by type, length* as covariate and random intercepts for *items, participants*, and *string-endings*. The same forward difference contrast coding scheme used in the previous analyses was set for *group* (see Appendix 1.2). As for *type*, we applied a forward difference contrast coding scheme comparing Novel Derivation vs. Real Suffix vs. Real Stem (see Appendix 2.2 for the coding matrix). Model comparison indicated that the interaction improved the model fit (*χ*^2^ = 231.45, p < .0001; see Appendix 2.3 for the comparison and 2.4 for the full interaction model), hence we proceeded to explore the pattern within each group by means of planned comparisons. We fitted four separate logit mixed effect models (one for each proficiency group) with *type* as predictor, string *length* as covariate, and random intercepts for *items, participants*, and *string-endings*.^13^ Full models are presented in Appendix 2.5.

Novel Derivations induced more errors (lowest rates of correct rejections) than Real Suffix pseudo-words for all proficiency groups (Beginner: OR = –1.36, *z* = –9.57, *p* < .001; Intermediate: OR = –2.34, *z* = –12.56, *p* < .001; Advanced: OR = –2.40, *z* = –10.71, *p* < .001; L1: OR = –3.05, *z* = –12.00, *p* < .001).^14^ However, Real Suffix pseudo-words induced less errors than Real Stem pseudo-words only for beginner (OR = 0.80, *z* = 3.72, *p* < .001) and intermediate (OR = 0.93, *z* = 3.15, *p* < .001) participants, while no significant difference was detected for advanced(OR = 0.4, *z* = 1.01, *p* = 0.313) and L1 (OR = –0.02, *z* = –0.04, *p* = 0.971) participants.

### 3.2 Analysis of reaction times

**Figure 2:**
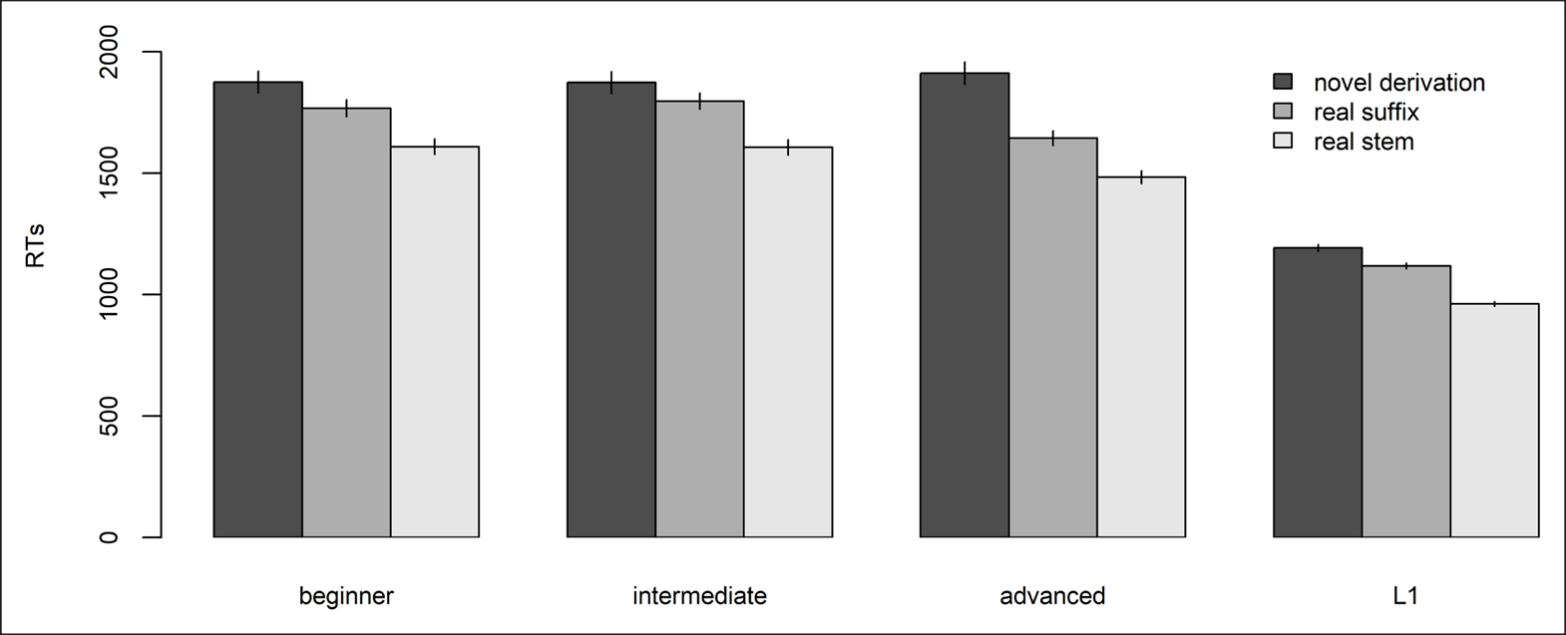
Mean reaction times for accurate rejections of different types of pseudo-words across groups. A table with detailed information is included in Appendix 3.1.

We then analyzed reaction times (RTs) for pseudo-word items. For the analyses of reaction times (RTs), we followed the approach outlined by Baayen & Milin (2010). First, we removed extreme outliers, that we defined here as datapoints exceeding 10000ms. A total of 160 datapoints (0.7% of the dataset) were identified as outlier and subsequently removed. Then we selected only response times associated to accurate answers (i.e., correct rejections). This further refinement led to the removal of 4,917 datapoints, which constituted 22.6% of the remaining dataset. The final dataset consisted of 16815 datapoints. Figure 2 plots mean RTs and standard error of the means for each group.

To correct for the intrinsic skewness of the variable, RTs for correct responses were log-transformed (Baayen & Milin, 2010). We then fitted two mixed-effects regression models (Baayen, Davidson, & Bates, 2008), one with *group, type*, and *length* as predictors, and random intercepts for *subjects, items*, and *string-ending*s, and one with a *group by type* interaction, *length* as predictor, and random intercepts for *subjects, items*, and *string-ending*s.^15^ A forward difference contrast coding scheme was applied to *group* and *type* following the same scheme as the previous analyses. Model comparison indicated that the interaction improved the model fit (*χ^2^* = 37.777, *p* < .001; see Appendix 3.2 for the full comparison). To exclude the impact of overly influential outliers, the model including the interaction was then refitted by removing datapoints with residuals exceeding 2.5 standard deviations (Baayen et al., 2008). The refitted model is reported in Appendix 3.3. The interaction between group and type was statistically significant (F(6, 16177.8) = 7.31, *p* < .001). To better understand the pattern of the interaction we fitted separate models for each proficiency group with *type* as predictor, string *length* as covariate, and random intercepts for *items, participants*, and *string-endings*. Models were then refitted by removing datapoints exceeding 2.5 standard deviations of the original interaction model (Baayen et al., 2008). Full refitted models are reported in Appendix 3.4.

The difference between Real Suffix and Real Stem pseudo-words was significant for all proficiency groups except for beginners (L1: β = 0.13, *t*(21.2) = 3.80, *p* < 0.001; Advanced: β = 0.08, *t*(28.02) = 2.19, *p* = 0.037; Intermediate: β = 0.08, *t*(29.59) = 2.55, *p* = 0.016; Beginner: β = 0.07, *t*(27.85) = 1.75, *p* = .09), with Real Stem pseudo-words generally rejected faster than Real Suffix pseudo-words. The comparison between Real Suffix pseudo-words and Novel Derivations was significant for all proficiency group (L1: β = 0.09, t(115.88) = 5.48, p < .001; Advanced: β= 0.17 *t*(132.32) = 6.58, *p* < .001; Intermediate: β = 0.11, *t*(130.7) = 4.33, *p* < .001; Beginner: β= 0.09, *t*(120.01) = 2.72, *p* = 0.007), with Novel Derivation yielding the longest reaction times.^16^

## 4. Discussion

In this study, we investigated the use of morphological information in the processing of pseudo-words and novel derivations and the effect of language proficiency. We compared L2 learners’ responses to pseudo-words (nonsense strings embedding a real stem *or* a real suffix) and novel derivations (novel combinations of existing stems and suffixes) in a lexical decision task. We were interested in assessing whether adult learners of a L2 would be influenced by the presence of morphological information when processing pseudo-words. Moreover, we were interested in investigating whether L2 learners would use morphological information productively when processing novel derivations. We hypothesized that, if L2 learners could use morphological information productively, then they should find it more difficult to classify novel derivations as non-existing words (in comparison to pseudo-words embedding only an existing stem or an existing suffix), since the combination of a stem and a suffix, even if unattested, could in principle be meaningful and interpretable. We were also interested in assessing the role of proficiency in the process. For this reason, we included in our study three groups of L2 learners, based on progressive language proficiency; we also included a L1 speakers control group to further track the impact of proficiency and to provide a comparison level to advanced learners.

Our results show that adult L2 learners were influenced by morphological information when processing pseudo-words and that, crucially, this pattern was modulated by language proficiency. In fact, while advanced learners and L1 speakers were similarly accurate in rejecting pseudo-words embedding an existing suffix vis-à-vis pseudo-words embedding an existing stem, beginner and intermediate level learners were more accurate in rejecting pseudo-words embedding an existing suffix than pseudo-words embedding an existing stem. This pattern suggests that participants with lower levels of proficiency relied on (pseudo-)stems rather than suffixes in order to classify pseudo-word items: the presence of a stem they recognized induced them to classify the pseudo-word as a word, thus leading to lower accuracy rates. In contrast, advanced learners and L1 participants accurately rejected both types of pseudo-words, suggesting that they had enough experience with the language to correctly select the appropriate response. However, for higher proficiency participants, rejecting a pseudo-word embedding a suffix proved to be more difficult than rejecting a pseudo-word embedding a stem. In fact, in terms of reaction times, pseudo-words embedding a stem were rejected more quickly than pseudo-words embedding a suffix for all proficiency groups except for beginner level learners. This pattern suggests that, particularly among learners with medium to high proficiency, the presence of a suffix poses greater challenges in rejecting pseudo-words. It indicates that as participants reach higher levels of proficiency, they become more sensible to the significance of morphological cues, specifically suffixes, when processing pseudo-words. Consequently, morphological information can interfere with the accurate classification of pseudo-words, leading to difficulties in correctly identifying them as such.

The activation of stems in pseudo-words without a morphological structure appears to be in line with the *embedded stem activation* account (e.g., Grainger & Beyersmann, 2017; Beyersmann et al., 2015; Beyersmann, Cavalli, Casalis & Colé, 2016). According to this account, stems hold a prominence in language processing and retain a prominent role in processing familiar and unfamiliar words. During language development, the activation of stems constitutes a first step in the acquisition of the morphological decomposition mechanism, which supports the decomposition of complex words (familiar and unfamiliar) into stems and affixes (Grainger & Beyersmann, 2017). However, at an early developmental stage, the morphological decomposition mechanism is not yet in place, therefore stems are always activated, also in the absence of a morphological structure (e.g., *cash* in *cash*ew; see also Hasenäcker, Solaja & Crepaldi, 2021). This account has also been extended to data from pseudo-word processing. A series of pseudo-word priming experiments with L1 participants showed that (pseudo-)stems are activated independently of the presence of an affix (Beyersmann et al., 2016; Hasenäcker et al., 2016). Similarly, in an unprimed lexical decision with L2 participants, Casalis et al. (2015) reported that pseudo-words embedding a stem (e.g., *foodle*) elicited longer reaction times than pseudo-words embedding a suffix in high and low proficiency groups. In line with this account, also in our data, beginner level participants relied more heavily on stems in their judgment, also in the absence of a morphological ending. However, while previous literature reports a prominence of stems also in high proficiency individuals, in our data, the relevance of morphological cues (i.e., suffixes) increased with language proficiency, effectively offsetting the relevance of stems, as indicated by differences in reaction times between Real Stem and Real Suffix pseudo-words emerging for medium to high level speakers (Intermediate, Advanced, and L1).

Consistent with the *embedded stem activation* account, our findings indicate a later emergence of affix representations. However, while this account posits that the importance of stems continues growing with language proficiency, guiding (novel) word processing also in proficient learners (e.g., Casalis et al., 2015; Beyersmann et al., 2016; Hasenäcker, Solaja & Crepaldi, 2020), our data highlight the prominent role of affixes in proficient learners. According to Beyersmann et al. (2016), stems become even more relevant for high proficiency readers because increased experience with a language allows individuals to easily map embedded stems on whole-word representations. We don’t see however why this explanation should be limited to stems and not be extended also to affixes. In fact, increasing language experience should also make individuals more sensitive to subtle regularities and statistical patterns in the language, which support the formation of morphological knowledge. Morphemes are non-other than recurrent patterns of form-meaning mapping in a language (e.g., Baayen, Milin, Đurđević, Hendrix, & Marelli, 2011; Marelli, Amenta & Crepaldi, 2015; Amenta, Günther & Marelli, 2020), and affixes are regular and frequent morphemes, which modify stems in predictable ways (Marelli & Baroni, 2015). Moreover, not only are affixes frequent and reliable linguistic units, but they are also highly informative: languages have only a restricted number of suffixes which can be combined with a large number of stems. The relative sparsity of affixes compared to stems makes the information carried by affixes more distinctive, hence salient. Indeed, it has been shown that morphological knowledge and awareness increases with language proficiency as individuals become more and more aware of and able to extract regularities from the linguistic continuum (e.g., Diependaele et al., 2011; Menutet al., 2022; Schmidtke et al., 2022). Therefore, it can be expected that with growing levels of proficiency, individuals learn to map not only stems but also affixes on their corresponding representation. Moreover, as affixes are frequent and reliable patterns, it is possible to assume that they would gain even more salience during processing. Our data support this view, showing that participants with higher levels of proficiency (Intermediate, Advanced and L1) demonstrate the ability to recognize and utilize suffixes as informative cues of lexicality, thus taking longer to reject a string embedding a suffix.

Taken altogether, results on pseudo-word processing reveal that low proficiency learners (beginner level participants) relied more on stems than affixes to classify pseudo-words, and that processing pseudo-words embedding a suffix did not require additional costs. High proficiency participants (advanced level learners and L1) showed similar accuracy rates for pseudo-words embedding stems and pseudo-words embedding suffixes, however, rejecting pseudo-words embedding suffixes required additional processing cost, indicating that these groups of participants relied more on morphological information (i.e., suffixes) to classify pseudo-words. Finally, intermediate level participants presented an in-between pattern: while, similarly to beginners, they were more accurate in rejecting pseudo-words embedding a suffix than pseudo-words embedding a stem, the rejection of pseudo-words embedding a suffix required additional cost, yielding longer reaction times. This pattern suggests that intermediate level learners still rely on stems to classify pseudo-words, however, given the higher linguistic experience they may have formed enough morphological knowledge to be influenced by the presence of a suffix. In conclusion, we can speculate that, for high proficiency learners, the presence of a pseudo-stem is a less salient cue for the existence of a word in comparison to the presence of a suffix.

Concerning novel derivations, our results showed that novel combinations of stems and suffixes were more difficult to reject, both in terms of accuracy and response times, than pseudo-words embedding only an existing suffix and pseudo-words embedding only an existing stem for all proficiency groups. This pattern is in line with previous literature on both L1 and L2 speakers (e.g., Longtin & Meunier, 2005; Beyersmann et al., 2015; Hasenäcker et al., 2016; Casalis et al., 2015). Previous research showed that both L1 speakers and L2 learners activate morphological information when processing novel derivations, i.e., unattested combination of real stems and suffixes: indeed, novel derivations elicited greater priming effects in experiments with L1 speakers (Longtin & Meunier, 2005; Beyersmann et al., 2015; Hasenäcker et al., 2016) than pseudo-words embedding either existing stems or suffixes. Moreover, in a lexical decision study with L2 learners, Casalis et al. (2015) reported that novel derivations required longer latencies to be rejected and showed lowest accuracy rates. The observed pattern corresponds to the *morpheme interference effect* (e.g., Taft & Forster, 1975; Crepaldi, Rastle & Davis, 2010; Beyersmann, Mousikou, Javourey-Drevet, Schroeder, Ziegler & Grainger, 2020), for which rejecting pseudo-words with a morphological structure (i.e., embedding a stem and an affix) requires longer reaction times in comparison to pseudo-words without a morphological structure. This effect has been explained in terms of the ability of the morphological structure of the pseudo-word to activate lexical representations corresponding to the (pseudo-)stem and the affix (Taft & Forster, 1975; Crepaldi et al., 2010). This account focuses on the impact of surface level information in processing pseudo-words: the mere presence of a possible stem *and* a possible affix (i.e., a morphological structure) is sufficient to hinder the recognition process, inducing a word response also for unattested strings. Interestingly, the more the novel combination of stem and affix is interpretable, the harder it is to reject it in a lexical decision task (Burani et al., 1999; 2002). The role of interpretability allows us to integrate the morphological account with semantic considerations. A legal combination of stem and affixes constitutes a novel word, and, it has been shown that, upon encountering a novel word, readers attempt meaning computation by means of a combinatorial process (Marelli & Baroni, 2015), hinting at a productive use of morphological information.

Looking at the full pattern of response accuracies, we can identify an effect of language proficiency in the productive use of morphology, that is, in the ability to attempt the combination of stems and affixes also in unknown instances. In particular, the pattern of responses in the pseudo-word items seems to be informative of different processes taking place in the processing of novel derivation as a function of linguistic proficiency. In our data, low proficiency participants (Beginner and Intermediate), showed a graded accuracy pattern from pseudo-words embedding a suffix (that were rejected quite accurately) to pseudo-words embedding a stem (which induced more mistakes) to novel derivations (for which accuracy was the lowest). Based on this pattern, it is then possible that the pseudo-word classification process was mostly guided by the recognition of a known stem. When both stems and suffixes were present, like in novel derivations, beginner and intermediate level participants made even more mistakes, but we can speculate that their process relied more on the presence of a familiar stem in a word-like context (enhanced by the presence of an affix) than on truly combinatorial processes. Of course, we cannot exclude that low proficiency participants may have attempted combinations of stems and affixes, but the high interference of stems and the relatively low interference of suffixes in pseudo-word recognition suggests that the process was guided predominantly by stem recognition. In fact, in order to trigger a real combinatorial process, it is necessary to extract information from both stem and affixes (e.g., Amenta et al., 2015), and we have evidence in our data that beginner and intermediate learners failed to appreciate the information conveyed by affixes. This observation aligns with existing literature on the acquisition of unfamiliar words, which suggests that learners with lower proficiency levels tend to depend on embedded stems as a means of determining the meaning of unfamiliar words (e.g., Bertram, Laine & Virkkala, 2000).

In contrast, advanced learners and L1 speakers were equally accurate in rejecting pseudo-words embedding a stem vis-à-vis pseudo-words embedding a suffix, suggesting that they had enough language experience to correctly understand that the string they were reading, even if it contained a known stem or a known suffix, were still nonexistent in the lexicon. Accuracy for these two types of pseudo-words reached almost ceiling levels (98% and 97% respectively). A different case was that of Novel Derivations, where the high number of inaccurate responses (due to a tendency to classify the novel derivation as words), can be attributed to an attempt of stem and affix combination. Only in this case, the presence of a stem *and* a suffix seems to trigger an attempt at interpreting the string through a real combinatorial process (Marelli & Baroni, 2015).

Overall, our results can be informative of the ability of L2 learners to exploit morphological information productively to process unattested combinations of word and affixes. Indeed, the role of morphological information is linked to language productivity: speakers of a language can create new labels for new concepts ex-novo, but they can also exploit morphology and morphological information to modify existing concepts or label new ones, as in the case of “petaloso” (Marelli & Baroni, 2015). The same process can also be observed during language processing: when readers encounter a word for the first time, they can rely on different cues in order to extract its meaning. One very strong cue is its structure: they can identify part of it as a string that they already know (pseudo-stem), or they can recognize an affix, and they can try to combine them in order to compute the meaning of the previously unknown string (e.g., Burani, Dovetto, Spuntarelli, & Thornton, 1999; Burani & Thornton, 2003; Traficante, Marcolini, Luci, Zoccolotti, & Burani, 2011). The role of affixation in novel derived forms is also supported by an expanding literature focusing on the ability of sub-lexical information to encode meaning (e.g., Marelli & Baroni, 2015; Hendrix & Sun, 2021; Gatti, Marelli & Rinaldi, 2022) and by computational models that correctly predict human semantic intuitions about novel word meaning based on morpheme combination (Marelli & Baroni, 2015; Günther & Marelli, 2020).

In conclusion, with this research, we have shown that morphological information is a strong cue for L2 word processing, and it is exploited to process unattested combinations of words and affixes (novel derivations). Most importantly, the ability to extract and exploit morphological information is linked to language proficiency: with language experience, not only the awareness of the morphological structure of words grows, but also the process of morphological decomposition and combination becomes more automatic (e.g., Diependaele et al., 2011; Dal Maso and Giraudo, 2014; Dawson et al., 2018; Deacon, Kieffer, & Laroche 2014; Jarmulowicz, Hay, Taran, & Ethington, 2008). In other words, it takes experience with a language in order to recognize meaningful patterns and use morphological information productively.

## Data Accessibility Statement

The data, code, and materials are available at https://osf.io/mpfdy/?view_only=7bc2f6e47e4c4cd98e4065c475f901fd.

## Additional File

The additional file for this article can be found as follows:

10.5334/joc.420.s1 Appendix. The Appendix provides details of the statistical models used and the complete results, such as coefficients, confidence intervals, and significance tests, supporting the main findings presented in the study.

## Footnotes

https://www.corriere.it/scuola/16_febbraio_24/ferrara-copparo-piccolo-matteo-inventa-parola-petaloso-accademia-crusca-risponde-7296e148-dac9-11e5-956c-6f7e55711737.shtml.

https://accademiadellacrusca.it/it/contenuti/la-parola-petaloso-ha-possibilita-di-entrare-nei-vocabolari/6376.

Since all the participants were officially enrolled in a university-related course of L2 Italian or in language schools, their proficiency level has been objectively assessed with official tasks and exams according to the CEFR. In this research we define learners with a proficiency level A as beginners, with a level B as intermediate, and with a level C as advanced.

Common European Framework of Reference for Languages. For a detailed description of proficiency levels in the CEFR see: https://www.coe.int/en/web/common-european-framework-reference-languages/leveldescriptions.

The high standard deviation in the L2 sample is mainly due to the recruitment locations. While participants coming from Het Perspectif ranged between 17 and 23 years of age, participants from SNT ranged between 24 and 71 years of age.

Some of the morphologically complex items in the current experiment were created by combining a stem (root + thematic vowel, e.g., *piovenza* where *piov-* is the root and *-e* the thematic vowel) and an affix (*-nza*), while in some cases the combination only included a root and an affix (e.g., *stelloso*, where *stell-* is the root and *-oso* the affix). To maintain clarity and consistency throughout the manuscript, we opted to always use the word “stem” to define the base morpheme in all our items, even if, in some cases, “root” would have been more appropriate.

Part of the data were collected relying on Pavlovia, an online platform who allows running experiments programmed in Psychopy2 (Bridges, Pitiot, MacAskill & Peirce, 2020).

Word frequency was extracted from SUBTLEX-IT (Crepaldi et al., 2015), while length was computed in characters.

We first run the model with random intercepts for items and participants and the random slope of group on items, the model, however failed to converge, even when an optimizer was used. We decided then to remove the random slope and keep only the random intercepts.

We assumed that beginners would be the group to produce the larger number of errors and have slower reaction times while L1 the group to produce the smallest number of errors and have fastest reaction times.

We first run the model with random intercepts for items and participants and the random slope of group on items. To facilitate convergence, we added the optimizer glmerControl (optimizer = c(“bobyqa”), optCtrl = list (maxfun = 100000000)). Despite this, the model failed to converge and thus we removed the random slope and only kept the random intercepts.

String-ending was included as a random intercept to control for the possible source of variance introduced by the morphological endings of Novel Derivations and Real Suffix pseudo-words. Non-morphological endings tend to be more salient in the lexicon and this could in principle impact the comparison with pseudo-words without a morphological ending.

The optimizer glmerControl (optimizer = c(“bobyqa”), optCtrl = list (maxfun = 100000000)) was added to all models to facilitate convergence.

In order to gather the comparison between Novel Derivation and Real Stem pseudo-words, we re-run the analyses with a difference contrast coding scheme comparing Novel Derivation vs. Real Stem vs. Real Suffix. Results indicated that Novel Derivations induced more errors than Real Stem pseudo-words for all proficiency groups (Beginner: OR = –0.56, z = –2.68, p = .007; Intermediate: OR = –1.41, z = –4.98, p < .001; Advanced: OR = –2.004, z = –5.25, p < .001; L1: OR = –3.07, z = –7.14, p<.001).

As before, we first ran the models including a random slope of group on items, but this resulted in failure of convergence. Thus, we removed the random slope and only kept the random intercepts.

To gather the comparison between Novel Derivation and Real Stem pseudo-words, we re-run the analyses with a difference contrast coding scheme for *type* comparing Novel Derivation vs. Real Stem vs. Real Suffix. Results indicated that Novel Derivations yielded longer reaction times than Real Stem pseudo-words for all proficiency groups (L1: β = 0.22, t(21.96) = 6.57, p < .001; Advanced: β = 0.25, t(31.49) = 6.48, p < .001; Intermediate: β = 0.196, t(35.86) = 5.77, p < .001; Beginner: β = 0.15, t(31.39) = 3.84, p < .001).
