# Supplementary material for: The Role of Morphological Information in Processing Pseudo-words in Italian L2 Learners: It’s a Matter of Experience
Source: J Cogn. 2025 Jan 7;8(1):14. doi: 10.5334/joc.420 (PMC11720858; doi:10.5334/joc.420)
Supplement: Appendix. — The Appendix provides details of the statistical models used and the complete results, such as coefficients, confidence intervals, and significance tests, supporting the main findings presented in the study. [file joc-8-1-420-s1.pdf]

Appendix

1. Word accuracy analyses

1.1 Mean accuracy and standard deviation of word trials

	Beginner	Intermediate	Advanced	L1
Morphologically complex	0.76 (0.14)	0.87 (0.05)	0.87 (0.06)	0.96 (0.04)
Morphologically simple	0.65 (0.17)	0.74 (0.09)	0.79 (0.08)	0.94 (0.05)
All	0.70 (0.15)	0.80 (0.06)	0.83 (0.06)	0.950.04)

1.2 Contrast coding scheme for the group variable

Group	1st contrast Beginner vs. Intermediate	2nd contrast Intermediate vs. Advanced	3rd contrast Advanced vs. L1
Beginner	0.75	0.5	0.25
Intermediate	-0.25	0.5	0.25
Advanced	-0.25	-0.5	0.25
L1	-0.25	-0.5	-0.75

1.3 Word accuracy: group

Model: glmer(resp_corr~group+ zipf + length +(1|item)+(1|sub),data=dataw_freq,family=binomial)

	**Accuracy Words**				
Predictors	*Estimate*	*std. Error*	*CI*	*z*	*p*
(Intercept)	0.04	0.03	0.01 – 0.21	-3.88	**<0.001**
Beginner vs. Intermediate	0.44	0.10	0.29 – 0.68	-3.77	**<0.001**
Intermediate vs. Advanced	0.74	0.16	0.49 – 1.11	-1.45	0.147
Advanced vs. L1	0.14	0.03	0.09 – 0.20	-9.65	**<0.001**
Frequency (zipf)	4.20	0.71	3.02 – 5.85	8.51	**<0.001**
Length	1.10	0.06	0.98 – 1.23	1.64	0.101
**Random Effects**					
σ^2	3.29				
$\tau_{00\ sub}$	0.63				
$\tau_{00\ item}$	1.84				
ICC	0.43				
N_{item}	98				
N_{sub}	146				
Observations	14302				
Marginal R^2 / Conditional R^2	0.342 / 0.625				

1.4 Word accuracy: Anova between models with and without type by group interaction

Models:

Data: dataw_freq

No interaction: glmer (resp_corr ~ group + type + zipf + length + (1 | item) + (1 | sub)

Interaction: glmer (resp_corr ~ group * type + zipf + length + (1 | item) + (1 | sub)

	npar	AIC	BIC	logLik	deviance	Chisq	Df	Pr(>Chisq)
No interaction	9	7992.5	8060.6	-3987.3	7974.5			
Interaction	12	7987.5	8078.3	-3981.8	7963.5	11.003	3	0.01171 *

1.5 Word accuracy: group by type

glmer(resp_corr~group * type + zipf + length +(1|item)+(1|sub),data=dataw_freq,family=binomial)

	**Word Accuracy**				
Predictors	*Estimate*	*std. Error*	*CI*	*Statistic*	*p*
(Intercept)	1.43	1.45	0.20 – 10.43	0.35	0.724
Beginner vs. Intermediate	0.31	0.08	0.20 – 0.50	-4.84	**<0.001**
Intermediate vs. Advanced	0.90	0.21	0.56 – 1.43	-0.45	0.650
Advanced vs. L1	0.13	0.03	0.08 – 0.21	-8.41	**<0.001**
type [simple]	0.14	0.05	0.07 – 0.30	-5.11	**<0.001**
Frequency (zipf)	3.94	0.60	2.93 – 5.31	9.04	**<0.001**
Length	0.83	0.06	0.71 – 0.96	-2.49	**0.013**
Group 1 × type [simple]	1.70	0.27	1.24 – 2.33	3.33	**0.001**
Group 2 × type [simple]	0.74	0.12	0.53 – 1.03	-1.80	0.071
Group 3 × type [simple]	1.11	0.22	0.74 – 1.65	0.49	0.623
**Random Effects**					
σ^2	3.29				
$\tau_{00\ sub}$	0.63				
$\tau_{00\ item}$	1.45				
ICC	0.39				
N_{item}	98				
N_{sub}	146				
Observations	14302				
Marginal R^2 / Conditional R^2	0.393 / 0.628				

1.6 Word Accuracy: planned comparisons (groups)

Models:
glmer(resp_corr~type+zipf+length+(1|item)+(1|sub),data=dataw_freq[dataw_freq$group=="beginner",],family=binomial)

The model was run four times on the subsets beginner, intermediate, advanced and L1.

	**Beginner**					**Intermediate**					**Advanced**					**L1**				
Predictors	*Estimate*	*std. Error*	*CI*	*z*	*p*	*Estimate*	*std. Error*	*CI*	*z*	*p*	*Estimate*	*std. Error*	*CI*	*z*	*p*	*Estimate*	*std. Error*	*CI*	*z*	*p*
(Intercept)	3.13	3.66	0.32 – 30.98	0.97	0.330	0.23	0.31	0.02 – 3.09	-1.11	0.269	0.23	0.38	0.01 – 5.61	-0.90	0.368	4.15	5.16	0.36 – 47.54	1.14	0.253
type [simple]	0.17	0.07	0.07 – 0.40	-4.11	**<0.001**	0.08	0.04	0.03 – 0.23	-4.85	**<0.001**	0.08	0.05	0.02 – 0.29	-3.91	**<0.001**	0.26	0.12	0.10 – 0.65	-2.87	**0.004**
zipf	2.29	0.40	1.63 – 3.22	4.79	**<0.001**	6.85	1.50	4.46 – 10.53	8.78	**<0.001**	10.70	3.06	6.11 – 18.74	8.30	**<0.001**	4.49	0.90	3.03 – 6.65	7.50	**<0.001**
length	0.80	0.07	0.68 – 0.95	-2.53	**0.011**	0.83	0.08	0.69 – 1.02	-1.79	0.073	0.75	0.09	0.59 – 0.96	-2.27	**0.023**	0.83	0.08	0.69 – 1.00	-1.94	0.052
**Random Effects**																				
σ^2	3.29					3.29					3.29					3.29				
τ_{00}	1.73 $_{item}$					2.09 $_{item}$					3.07 $_{item}$					1.32 $_{item}$				
	0.99 $_{sub}$					0.43 $_{sub}$					0.73 $_{sub}$					0.78 $_{sub}$				
ICC	0.45					0.43					0.54					0.39				
N	98 $_{item}$					98 $_{item}$					98 $_{item}$					98 $_{item}$				
	28 $_{sub}$					35 $_{sub}$					32 $_{sub}$					51 $_{sub}$				
Observations	2744					3424					3136					4998				
Marginal R^2 / Conditional R^2	0.136 / 0.527					0.390 / 0.654					0.420 / 0.731					0.270 / 0.554				

2 Pseudo-word accuracy

2.1 Mean accuracy (correct rejections) and standard deviation of non-word trials.

	Beginner	Intermediate	Advanced	L1
Real Stem	0.60 (0.22)	0.80 (0.13)	0.90 (0.08)	0.98 (0.04)
Real Suffix	0.71 (0.19)	0.86 (0.1)	0.91 (0.1)	0.97 (0.04)
Novel derivation	0.46 (0.22)	0.47 (0.16)	0.59 (0.14)	0.73 (0.14)

2.2 Contrast coding scheme for the type variable

Type	1st contrast<br>Novel Derivation vs. Real Suffix	2nd contrast<br>Real Suffix vs. Real Stem
Novel Derivation	0.67	0.33
Real Suffix	-0.33	0.33
Real Stem	-0.33	-0.67

2.3 Anova between models with and without interaction

Models:

Data: datanw

No interaction: resp_corr ~ group + type + length + (1 | item) + (1 | sub) + (1 | ending)

Interaction: resp_corr ~ group * type + length + (1 | item) + (1 | sub) + (1 | ending)

	npar	AIC	BIC	logLik	deviance	Chisq	Df	Pr(>Chisq)
No interaction	10	16783	16863	-8381.5	16763			
Interaction	16	16564	16691	-8265.7	16532	231.45	6	< 001 ***

2.4 Pseudo-word accuracy: group by type

glmer(resp_corr~group*type+ length +(1|item)+(1|sub)+(1|ending),data=datanw,family=binomial)

	Pseudo-word Accuracy				
Predictors	*Estimate*	*std. Error*	*CI*	*Statistic*	*p*
(Intercept)	18.03	9.27	6.58 – 49.42	5.62	**<0.001**
Beginner vs. Intermediate	0.51	0.12	0.32 – 0.81	-2.86	**0.004**
Intermediate vs. Advanced	0.49	0.11	0.31 – 0.77	-3.13	**0.002**
Advanced vs. L1	0.24	0.05	0.16 – 0.38	-6.40	**<0.001**
Novel Derivation vs. Real Suffix	0.11	0.02	0.08 – 0.15	-14.67	**<0.001**
Real Suffix vs. Real Stem	1.73	0.46	1.03 – 2.90	2.08	**0.037**
Length	0.88	0.05	0.79 – 0.98	-2.31	**0.021**
Group1 × Type1	2.37	0.31	1.84 – 3.06	6.63	**<0.001**
Group2 × Type1	0.99	0.14	0.75 – 1.31	-0.04	0.965
Group3 × Type1	1.80	0.32	1.27 – 2.54	3.33	**0.001**
Group1 × Type2	1.15	0.15	0.89 – 1.49	1.08	0.282
Group2 × Type2	1.37	0.22	1.01 – 1.87	2.00	**0.045**
Group3 × Type2	1.42	0.32	0.92 – 2.20	1.57	0.117
**Random Effects**					
σ^2	3.29				
$\tau_{00\ item}$	0.48				
$\tau_{00\ sub}$	0.81				
$\tau_{00\ ending}$	0.15				
ICC	0.30				
N_{item}	150				
N_{sub}	146				
N_{ending}	25				
Observations	21892				
Marginal R^2 / Conditional R^2	0.339 / 0.540				

2.4 Pseudo-word accuracy: planned comparisons (groups)

```
glmer(resp_corr~type+length+(1|item)+(1|sub)+(1|ending),data=datanw[datanw$group=="beginner
",],family=binomial)
```

The model was run four times on the subsets beginner, intermediate, advanced and L1.

	**Beginner**					**Intermediate**					**Advanced**					**L1**				
Predictors	*Estimates*	*std. Error*	*CI*	*z*	*p*	*Estimates*	*std. Error*	*CI*	*z*	*p*	*Estimates*	*std. Error*	*CI*	*z*	*p*	*Estimates*	*std. Error*	*CI*	*z*	*p*
(Intercept)	3.65	1.83	1.37 – 9.73	2.59	**0.009**	20.41	12.53	6.13 – 67.97	4.91	**<0.001**	30.77	23.70	6.80 – 139.28	4.45	**<0.001**	73.50	62.53	13.87 – 389.41	5.05	**<0.001**
Novel Derivation vs. Real Suffix	0.26	0.04	0.19 – 0.34	-9.57	**<0.001**	0.10	0.02	0.07 – 0.14	-12.56	**<0.001**	0.09	0.02	0.06 – 0.14	-10.71	**<0.001**	0.05	0.01	0.03 – 0.08	-12.00	**<0.001**
Real Suffix vs. Real Stem	2.23	0.48	1.46 – 3.41	3.72	**<0.001**	2.54	0.75	1.42 – 4.54	3.15	**0.002**	1.49	0.59	0.69 – 3.24	1.01	0.313	0.98	0.45	0.40 – 2.39	-0.04	0.971
length	0.92	0.04	0.84 – 1.01	-1.72	0.085	0.82	0.05	0.72 – 0.93	-3.02	**0.003**	0.86	0.07	0.74 – 1.01	-1.82	0.068	0.92	0.08	0.77 – 1.10	-0.87	0.385
**Random Effects**																				
σ^2	3.29					3.29					3.29					3.29				
τ_{00}	0.29_{item}					0.62_{item}					0.86_{item}					0.90_{item}				
	1.31_{sub}					0.57_{sub}					0.64_{sub}					0.99_{sub}				
	0.06_{ending}					0.14_{ending}					0.34_{ending}					0.40_{ending}				
ICC	0.34					0.29					0.36					0.41				
N	150_{item}					150_{item}					150_{item}					150_{item}				
	28_{sub}					35_{sub}					32_{sub}					51_{sub}				
	25_{ending}					25_{ending}					25_{ending}					25_{ending}				
Observations	4200					5242					4800					7650				
Marginal R^2 / Conditional R^2	0.060 / 0.375					0.189 / 0.422					0.201 / 0.487					0.285 / 0.578				

3. Pseudo-word reaction time analyses

3.1 Mean reaction time and standard error of the mean of non-word trials

	Beginner	Intermediate	Advanced	L1
Real Stem	1609.66 (32.27)	1607.25 (31.35)	1484.38 (26.63)	962.26 (9.28)
Real Suffix	1768.27 (35.31)	1796.91 (33.49)	1645.11 (30.51)	1118.69 (12.03)
Novel derivation	1875.80 (44.52)	1874.11 (45.77)	1912.39 (46.07)	1192.61 (14.08)

3.2 Anova between models with and without interaction

Models:

Data: cutnw1

No interaction: log(resp_rt) ~ group + type + length + (1 | item) + (1 | sub) + (1 | ending)

Interaction: log(resp_rt) ~ group * type + length + (1 | item) + (1 | sub) + (1 | ending)

	npar	AIC	BIC	logLik	deviance	Chisq	Df	Pr(>Chisq)
No interaction	11	13257	13342	-6617.7	13235			
Interaction	17	13232	13363	-6598.8	13198	37.777	6	< 001 ***

3.3 Full model with interaction type by group

Model: lmer(log(resp_rt)~group*type+length+(1|item)+(1|sub)+(1|ending),data=cutnw1, subset=abs(scale(resid(i)))<2.5))

	Sum Sq	Mean Sq	NumDF	DenDF	F value	Pr(>F)
Group	4.2618	1.4206	3	142.3	15.2264	1.203e-08 ***
type	10.0312	5.0156	2	60.4	53.7583	3.884e-14 ***
length	15.0054	15.0054	1	143.0	160.8323	< 2.2e-16 ***
group:type	4.0937	0.6823	6	16177.8	7.3129	8.013e-08 ***

3.4 Pseudo-word reaction times: paired comparisons

Model:
lmer(log(resp_rt)~type+(1|item)+(1|sub)+(1|ending),data=cutnw1[cutnw1$group=="beginner",])

Subset: abs(scale(resid(i2))) < 2.5

The model was run four times on the subsets beginner, intermediate, advanced and L1.

	**Beginner**					**Intermediate**					**Advanced**					**L1**				
Predictors	*Estimates*	*std. Error*	*CI*	*t*	*p*	*Estimates*	*std. Error*	*CI*	*t*	*p*	*Estimates*	*std. Error*	*CI*	*t*	*p*	*Estimates*	*std. Error*	*CI*	*t*	*p*
(Intercept)	7.38	0.06	7.26 – 7.51	118.80	**<0.001**	7.34	0.07	7.20 – 7.48	102.87	**<0.001**	7.27	0.07	7.13 – 7.41	101.82	**<0.001**	6.91	0.04	6.83 – 7.00	159.65	**<0.001**
Novel Derivation vs. Real Suffix	0.09	0.03	0.02 – 0.15	2.72	**0.007**	0.11	0.03	0.06 – 0.17	4.33	**<0.001**	0.17	0.03	0.12 – 0.22	6.59	**<0.001**	0.09	0.02	0.06 – 0.13	5.48	**<0.001**
Real Suffix vs. Real Stem	0.07	0.04	-0.01 – 0.14	1.75	0.09	0.08	0.03	0.02 – 0.14	2.55	**0.011**	0.08	0.04	0.01 – 0.16	2.19	**0.037**	0.13	0.03	0.06 – 0.19	3.80	**<0.001**
**Random Effects**																				
σ^2	0.15					0.16					0.16					0.07				
τ_{00}	0.01_{item}					0.01_{item}					0.01_{item}					0.01_{item}				
	0.10_{sub}					0.17_{sub}					0.15_{sub}					0.08_{sub}				
	0.00_{ending}					0.00_{ending}					0.00_{ending}					0.00_{ending}				
ICC	0.43					0.54					0.51					0.57				
N	150_{item}					150_{item}					149_{item}					150_{item}				
	28_{sub}					35_{sub}					32_{sub}					51_{sub}				
	25_{ending}					25_{ending}					25_{ending}					25_{ending}				
Observations	2351					3621					3733					6675				
Marginal R^2 / Conditional R^2	0.013 / 0.440					0.016 / 0.545					0.028 / 0.526					0.046 / 0.592				
